# Characterization of NiCuO_x_N_y_ Coatings Obtained via RF Sputtering: Structure, Morphology, and Optical Properties

**DOI:** 10.3390/ma17133264

**Published:** 2024-07-02

**Authors:** Karen Lizzette Velásquez-Méndez, José Edgar Alfonso, Manuel Bethencourt, Gustavo Cifredo, Gloria Ivonne Cubillos

**Affiliations:** 1 Grupo de Materiales y Procesos Químicos, Departamento de Química, Universidad Nacional de Colombia, Av. Cra. 30 No 45-03, Bogotá 16486, Colombia; klvelasquezm@unal.edu.co (K.L.V.-M.); gcubillos@unal.edu.co (G.I.C.); 2 Grupo de Ciencia de Materiales y Superficies, Departamento de Física, Universidad Nacional de Colombia, Av. Cra. 30 No 45-03, Bogotá 14490, Colombia; jealfonsoo@unal.edu.co; 3 Department of Materials Science, Metallurgical Engineering and Inorganic Chemistry, Institute of Marine Science (INMAR), University of Cadiz, Polígono del Rio San Pedro s/n, 11510 Puerto Real, Spain; gustavo.cifredo@uca.es

**Keywords:** NiCuO_x_N_y_ films, band gap, semiconductor

## Abstract

The rapid advancement of technology necessitates the continual development of versatile materials that can adapt to new electronic devices. Rare earth elements, which are scarce in nature, possess the set of properties required for use as semiconductors. Consequently, this research aims to achieve similar properties using materials that are abundant in nature and have a low commercial cost. To this end, nickel and copper were utilized to synthesize thin films of nickel–copper binary oxynitride via reactive RF sputtering. The influence of nitrogen flow on the structure, morphology, chemical composition, and optical properties of the films was investigated using various characterization techniques, including X-ray diffraction (XRD), scanning electron microscopy (SEM), atomic force microscopy (AFM), and X-ray photoelectron spectroscopy (XPS), as well as transmittance and absorbance measurements. The crystalline structure of the films shows that they can have preferential growth or be polycrystalline according to the nitrogen flow used during deposition and that both the oxides and oxynitrides of metals are formed. We identified unknown phases specific to this material, termed “NiCuO_x_N_y_”. The morphology revealed that the grain size of the coatings was dependent on the nitrogen flow rate, with grain size decreasing as the nitrogen flow rate increased. Notably, the coatings demonstrated transparency for wavelengths exceeding 1000 nm, with an optical band gap ranging from 1.21 to 1.86 eV.

## 1. Introduction

The electromagnetic transparency exhibited by materials like transparent conductive oxides (TCO) across various regions of the electromagnetic spectrum has facilitated their utilization in diverse industrial applications, including smart windows, solar cells, high-emission diodes, and liquid crystal displays [1,2]. Among the wide array of TCO coatings, there are n-type wide-bandgap oxides such as Al-doped zinc oxide (Al-ZnO), indium tin oxide (ITO), Sb-doped SnO_2_, and In_2_O_3_ [3]. Nonetheless, certain elements like indium pose significant toxicity concerns and are costly, primarily due to limited production sources [4], underscoring the necessity for non-toxic, cost-effective alternative materials exhibiting similar or enhanced properties.

Copper and nickel, along with their oxides, find extensive use in electronic devices and solar cells owing to their affordability and favorable electrical conductivity, thus presenting themselves as potential substitutes for the aforementioned materials [5]. Materials based on these metals could offer a viable alternative. However, the scope of TCO applications is constrained by the fact that most are n-type semiconductors, which, despite their efficiency, come with a high cost. Consequently, recent research endeavors have focused on exploring p-type semiconductors. Copper oxides (CuO, Cu_2_O) and nickel oxide (NiO) are examples of p-type semiconductors [6] exhibiting high optical transmittance and low electrical resistivity, making them suitable candidates for the development of photoelectronic devices like solar cells and transparent electrodes.

Additionally, Cu-based oxides and Ni-based oxides exhibit remarkable chemical stability across all concentration ranges, suitable crystallinity, and possess wide direct energy band gaps: NiO (>3.0 eV), CuO (1.2–2.6 eV) [6,7], and Cu_2_O (2.1–2.6 eV) [8]. Moreover, Cu_2_O demonstrates a significant exciton binding energy (150 meV), enabling the observation of well-defined excitonic characteristics in the absorption spectrum at low temperatures [9]. Its high optical absorption coefficient within the visible range (350–800 nm) and favorable electrical properties make it suitable for thin film solar cell fabrication, with a theoretically achievable efficiency of up to 13%. Furthermore, Cu_2_O holds promise for applications in photochemistry, serving as a catalyst for water splitting. Conversely, the electro-optic behavior of nickel oxide varies depending on the atomic Ni/O ratio, influenced by Ni vacancies and/or interstitial oxygen in the NiO structure, leading to alterations in the absorption edge of the NiO coating. These variations impact the applications of NiO, including its use as an electrochromic material [10], solar cell electrodes [11], chemical gas sensors [12], and organic light-emitting diodes [13]. There are chemical and physical techniques to grow thin films of copper and nickel oxides, such as chemical bath deposition, dipping, spin coating [14], the sol–gel method [15], chemical spray pyrolysis [16], sputtering, and pulsed-laser deposition [17].

In this study, we determined new crystallographic structures of Ni-Cu coatings deposited in oxygen and nitrogen atmosphere via RF-magnetron sputtering, which were called NiCuON. The optical response of these coatings was analyzed, and a potential application as an optical filter in the UV electromagnetic spectrum was found.

## 2. Materials and Methods

### 2.1. Deposition of NiCuO_x_N_y_ Coatings

NiCuO_x_N_y_ films were deposited on standard glass substrates using RF reactive magnetron sputtering with Alcatel equipment model HS 2000 (Alcatel Vacuum Technology, Annecy, France). The device comprises an unbalanced magnetron operating at an RF frequency of 13.56 MHz, a vacuum chamber equipped with both mechanical and turbomolecular vacuum pumps, a gas mixer, a gas flow meter, and pressure control mechanisms. Gas flow was regulated and monitored using a mass flow controller and a capacitance manometer (MKS), respectively [18]. The chamber underwent evacuation to a base pressure of 1.0 × 10^–3^ Pa for 4 h, initially with the introduction of Ar (99.999%, Linde, Colombia) until reaching a pressure of 1.0 × 10^−1^ Pa, followed by the introduction of nitrogen and oxygen to achieve a working pressure of 7.4 × 10^−1^ Pa. This procedure ensured the removal of residual gases. Gas pressure was continuously monitored during each sputtering deposition to keep it constant, as the sputtering current is highly sensitive to pressure fluctuations. Deposition was carried out over 60 min using a copper (99.9%) target with a diameter of 8.0 cm and an exposed zone area of 0.60 cm thick, along with a nickel (99.9%) piece, 3.0 cm in diameter and 1.5 mm thick, placed atop the Cu target. This setup yielded a Ni versus Cu exposed surfaces ratio of 14%.

Throughout the deposition process, the working pressure remained at 7.4 × 10^–1^ Pa, with a working gas mixture of Ar (20.0 sccm) and O_2_ (2.00 sccm). To evaluate the effect of nitrogen flow rate on the structural, morphological, and optical properties of NiCuO_x_N_y_ coatings, the nitrogen flux was varied from 4.00 to 18.0 sccm. The power applied to the target and the deposition temperature were set at 250 W and 433 K, respectively. The distance between the target and the substrate was maintained at 5.0 cm. Depending on the deposition conditions, film thickness ranged from 2.43 to 4.30 µm, measured using a Veeco–Dektak 150 surface profilometer (Veeco Instruments Inc., Plainview, NY, USA). Table 1 summarizes the deposition conditions employed in this study.

### 2.2. Characterization Techniques

Structural analysis of the films was conducted using X-ray diffraction (XRD) on a Philips PANalytical X’PERT Pro diffractometer (PANalytical, Almelo, The Netherlands) with Cu-Kα radiation (λ = 0.1542 nm) in Bragg–Brentano configuration. X-ray patterns were obtained using a diffraction angle between 10° to 90°, with a step size of 0.02 degrees, at an accelerating voltage of 45 kV and an emission current of 40 mA. Phases were identified based on the Joint Committee on Powder Diffraction Standards (JCPDS) cards. Morphological analysis was carried out using an FEI Quanta scanning electron microscope (FEI Company, Hillsboro, OR, USA) equipped with an energy-dispersive X-ray (SEM-EDX) microprobe operating at 30 kV. Micrographs were captured at 2000× magnification.

Atomic force microscopy (AFM) was employed to determine the roughness parameters of deposited NiCuOxNy coatings. AFM measurements were performed using a Park Scientific Instruments AutoProbe CP (Park Systems, Suwon, Republic of Korea) instrument in non-contact mode with a 10 nm radius, a study area of 25 μm^2^, and a transmission frequency of 10 Hz. Surface chemical composition was analyzed using X-ray photoelectron spectroscopy (XPS) on a Specs spectrometer using a PHOIBOS 150 1D-DLD hemispherical energy analyzer (SPECS Surface Nano Analysis GmbH, Berlin, Germany) coupled with a differentially pumped electrostatic pre-lens system. The spectrometer is equipped with an XR-50 MF Al X-ray source and a μ-FOCUS 600 monochromator with an energy resolution better than 0.5 eV, operating under a vacuum greater than 3.0 × 10^–7^ Pa. Al Kα radiation (1486.6 eV) was used, with a constant pass energy of 100 eV for wide-scan spectra and 20 eV for narrow-scan spectra, yielding a calculated energy resolution of 0.50 eV. A full width at half maximum (FWHM) of 0.51 eV for the Ag 3d5/2 core level was measured. In the spectra measured as received in the laboratory, without any special pre-treatment, no evidence of N1s was found, and therefore, it was necessary to clean the surface with Ar+ ions. Prior to analysis, the specimen was sputtered with 3 keV Ar+ ions for 3.0 min, and depth profile spectra were registered. A 100 µm diameter analysis spot was used. The relative elemental compositions were calculated based on the area under the curve for each element’s XPS signal: Cu2p_3/2_, Cu2p_1/2_, Ni2p_3/2_, Ni2p_1/2_, N1s, and O1s peaks. Although some authors consider that using adventitious carbon C1s for charge correction is not advisable because it does not necessarily make electrical contact with the sample, uncertainty could be generated with the reported results [19,20,21], adventitious C1s presents sp^2^ hybridization, and the presence of π electrons generate charge δ that favors the interaction with the sample surface -C=C- [22]. The spectra were calibrated based on the C 1s sp^2^ hybridized electrons at 284.6 eV, with a ±0.2 eV accuracy in the binding energy (BE) measurements [23,24]. Background subtraction was performed using the Shirley algorithm binding energy. Peak fitting was performed using the χ^2^ minimization and optimization by Newton’s method, as implemented in the XPS-peak software XPSPEAK 4.1 (developer Raymund Kwow). The optical response was assessed through transmittance measurements spanning 200 to 2500 nm on a UV-Vis-NIR spectrophotometer Varian Cary 5000 (Agilent Technologies, Santa Clara, CA, USA) at room temperature (RT). The refractive index (η) as a function of wavelength was determined using the Swanepoel method [25,26].

## 3. Results and Discussion

### 3.1. Chemical Composition

Transition metal oxynitrides are characterized by the partial replacement of oxygen within the crystalline structure of the oxide by nitrogen through a solid-phase diffusion reaction. They can also form through the diffusion of oxygen into the nitride structure. This results in the properties of an ionic–covalent material with anion vacancies [27,28]. Depending on the degree of oxygen substitution by nitrogen, X-ray photoelectron spectroscopy will record the binding energies for the oxide/oxynitride or nitride/oxynitride mixture [29].

Transition metal oxynitrides are characterized by the substitution of part of the oxygen within the crystalline structure of the oxide with nitrogen through a solid-phase diffusion reaction. They also form through the diffusion of oxygen into the nitride structure. This gives them the characteristics of an ionic–covalent material with anion vacancies. Depending on the degree of oxygen substitution by nitrogen, X-ray photoelectron spectroscopy will record the binding energies for the oxide/oxynitride (Me-O, Me-O-N) or nitride/oxynitride (Me-N, Me-O-N) mixture.

**Figure 1:**
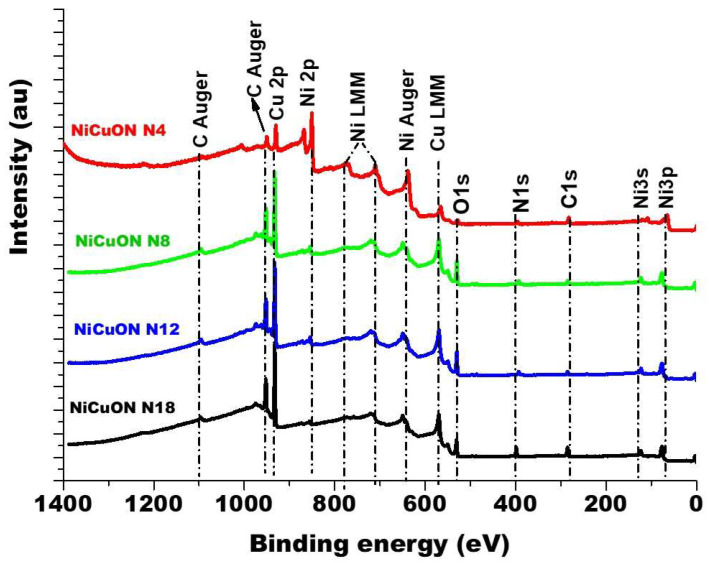
The survey XPS spectrum from thin films NiCuO_x_N_y_.

**Figure 2:**
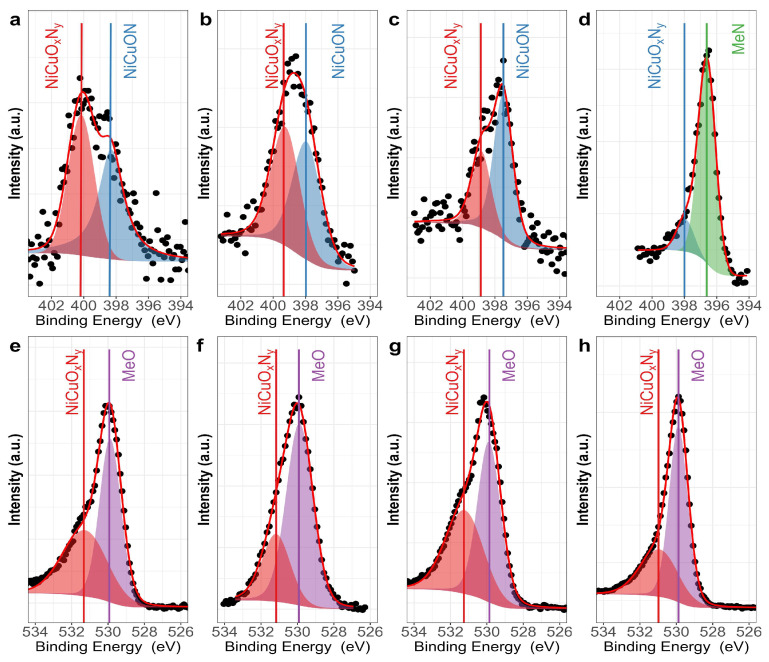
High-resolution XPS spectra recorded from NiCuO_x_N_y_, after argon etching for 3 min, for N1s (**a**–**d**) and O1s (**e**–**h**) core levels.

An analysis of the chemical composition of the coatings using X-ray photoelectron spectroscopy (XPS) was conducted to gather information regarding changes in surface chemical composition with varying nitrogen flux. During the analysis, the oxygen flow was maintained at a constant rate of 2.00 sccm, while the nitrogen flow was incrementally increased from 4.00 to 18.0 sccm. Under the different deposition conditions and directly on the surface, the only components of the film are the oxides NiO and CuO. After 3.0 min of etching in Ar+ atmosphere beam energy of 3 keV, the coatings, deposited at different nitrogen fluxes, exhibited varying compositions of Cu^1+^, Ni^2+^, O^2−^, and N^3−^. Figure 1 presents the survey spectrum for NiCuO_x_N_y_ films deposited with different nitrogen fluxes. The spectra show evidence of C, N_2_, O_2_, Cu, and Ni. Figure 2 shows high-resolution spectra corresponding to N1s and O1s core levels for the sample after sputtering in an argon atmosphere for 3.0 min. The deconvolution process applied to all peaks involved subtracting a Shirley-type baseline. Peak fitting was performed using the χ^2^ minimization and optimization by Newton’s method, as implemented in the XPS-peak software.

**Figure 3:**
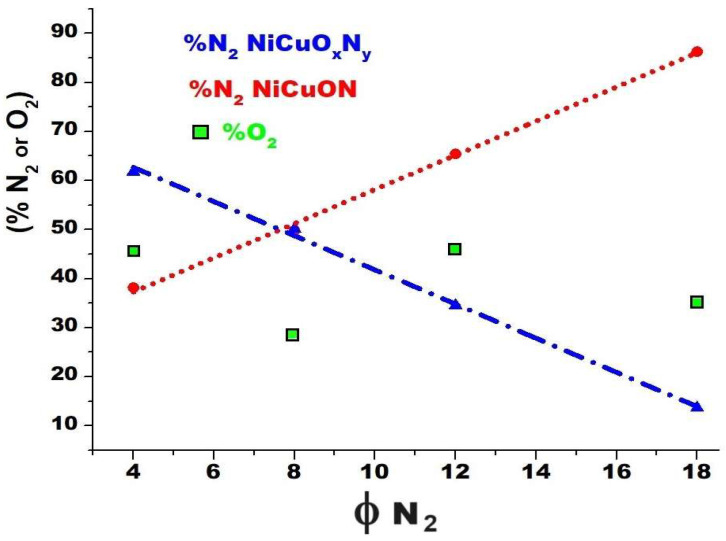
Composition of species obtained from the area under the curve for XPS spectra recorded from NiCuOxNy after argon etching for 3 min.

In Figure 2a–d, the N1s of the XPS spectra reveal two distinct bonding environments for nitrogen, which were deconvoluted into two oxynitride phases with differing degrees of nitrogen substitution. The predominantly oxygenated chemical species located at 400–399 eV, associated with MeO_x_N_y_, where Me = Cu or Ni [30], with a decreasing composition according to the nitrogen flow used during the film deposition: ϕN_2_(4) = 61.8%, ϕN_2_(8) = 50.1%, ϕN_2_(12) = 34.5%, and ϕN_2_(18) = 13.7% (Figure 3). The second peak, located at 398.4–397.5 eV, was associated with fewer oxygen species of oxynitride, MeON, with a composition that is a direct function of the nitrogen flow: ϕN_2_(4) = 38.2%, ϕN_2_(8) = 49.8%, and ϕN_2_(12) = 65.5%, to transform into the corresponding nitride (MeN) when the nitrogen flow reaches 18.0 sccm (Figure 3). It is observed that as the nitrogen flow increases, there is a shift toward lower binding energies (indicative of a greater reducing zone), suggesting a higher degree of oxygen substitution by nitrogen. The film deposited with a nitrogen flow of 18.0 sccm exhibited a different phase composition (Figure 2d). The N1s spectra signals displayed two peaks with different relative intensities, the first located at 398.0 eV with a chemical composition of 13.7% associated with the NiCuON species and the second at 396.6 eV with 86.3% potentially associated with the Ni–N and Cu–N bonds, as suggested by the literature data. These signals have been previously attributed by various authors to Me_x_N_y_ [26,27,28,29,30], possibly related to the corresponding nitride of NiCuN_y_, where copper would have a valence of 1+ and nickel 2+. However, assigning the binding energy for each metal is challenging, as both metals have similar electronegativities (Cu = 1.90 and Ni = 1.91) [31,32]. In this film, the nitrogen flow favored greater substitution of oxygen by nitrogen, facilitating nitride formation.

In general, it is observed in Figure 3 that the formation of the NiCuO_x_N_y_ species is a direct function of the nitrogen flow and that the concentration of NiCuO_x_N_y_ decreases with its increase, favoring the substitution of oxygen for nitrogen. For oxygen, a specific trend is not observed since it is associated with the formation of oxide and oxynitride.

For O1s, as shown in Figure 2e–h for the four studied nitrogen fluxes, two distinct chemical environments with varying relative compositions were confirmed. O1s at a binding energy of 529.9 eV, with a variable composition according to the deposit conditions, according to various authors, may be attributed to MeO [31,33]. O1s at a binding energy of 531.3 eV, with a relative composition of ϕN_2_(4) = 45.5% of O_2_, ϕN_2_(8) = 28.1% of O_2_, ϕN_2_(12) = 46.1% of O_2,_ and ϕN_2_(18) = 35.1% of O_2_, may be associated with NiCuO_x_N_y_ [30,31,32]. O1s located at 532.0 eV are often assigned to chemisorbed oxygen or surface water oxygen by various authors [34]. However, the spectrum was taken after a three-minute Ar^+^ cleaning, and physisorbed H_2_O was removed from the surface, and therefore, it corresponds to a different type of Me–O–N interaction, possibly indicating a form of oxynitride with higher oxygen content. Similar binding energies have been reported for zirconium oxynitrides [31,33], niobium oxynitride [34], and cerium oxynitride, among others [35,36].

**Figure 4:**
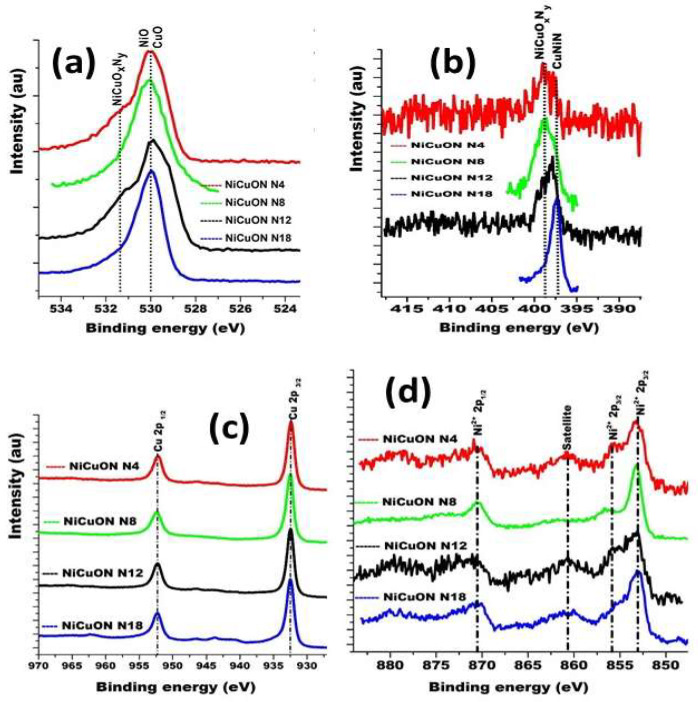
High-resolution XPS spectra recorded from NiCuO_x_N_y_, after argon etching for 3 min, for N_2_1s (**a**), N1s (**b**), Cu2p (**c**), and Ni2p (**d**) core levels.

Considering the Gibbs energies of copper oxide (−147.8 kJ·mol^−1^) and nickel oxide (−211 kJ·mol^−1^), the most likely outcome is the formation of NiO on the coatings’ surface under atmospheric pressure. However, chemical reactivity changes under low pressures. Results indicate that nitrogen and oxygen at 10^−2^ Pa exhibit similar chemical reactivity, suggesting the formation of copper oxide, nickel oxide, and copper–nickel oxynitrides. The oxygen composition of the coatings at different nitrogen fluxes is presented in Figure 4a. No significant changes in phase composition were observed for coatings deposited at varying nitrogen flows, suggesting that Gibbs’s energy for the growth of nickel–copper oxynitrides is independent of nitrogen flux.

In Figure 4b, spectra have been superimposed to assess changes in the chemical composition from the increase in the nitrogen flow. The results showed that the increase in the nitrogen produced on the surface predominates the NiCuN phase. This result can be explained by the fact that the nitrogen substitutes an oxygen in the crystalline structure. For copper (Figure 4c), the only consistently identified phase across all four N_2_/O_2_ flow ratios was Cu^1+^. The observed binding energy values could be attributed to Cu (I) bonded to O as Cu_2_O. These binding energy values have been reported for Cu_2_O nanoparticles synthesized using various methods [37,38,39,40,41,42], including Cu_2_O nanoparticles utilized for adsorption and CO_2_ photoreduction (Cu_2_O/TiO_2_) [37,40,41], Cu_2_O nanoparticles deposited on ZnO nanorods, and in thin films of cuprous oxide [40].

The Ni spectrum (Figure 4d) displays a spin-orbital doublet with multiple peaks, making interpretation challenging. However, the splitting observed between 853 and 856 eV is characteristic of Ni^2+^, as confirmed by the binding energies for Ni 2p_3/2_ and Ni 2p_1/2_, located at 853.1 eV and 870.5 eV, respectively, with a separation of 17.4 eV. In this region (856 eV), the sample deposited with a nitrogen flow of 8.00 sccm exhibits a 1.0 eV shift in binding energy compared to films deposited at N4, N12, and N18 sccm, while the binding energy at 870 eV remains unchanged, indicating the same oxidation state [42,43,44,45,46,47]. These results are consistent with those found via XRD, which indicate the presence of NiO and Cu_2_O_m_ phases in the same sample, suggesting that the last 10 nm of the coatings consist of copper and nickel oxides and oxynitrides.

An approximation of the chemical formula is presented in Table 2. However, an unequivocal assignment of the chemical formula is often difficult and controversial based solely on XPS data, considering the scarcity of literature data for the chemical species NiCuO_x_N_y_. The N/O ratio indicates a decrease in the degree of oxygen replacement by nitrogen with increasing nitrogen concentration in the medium. This phenomenon could be attributed to surface saturation, as oxynitride formation occurs through a solid-phase diffusion process. Similar observations have been made during the synthesis of oxynitride phosphate glasses [36].

### 3.2. Crystal Structure

**Figure 5:**
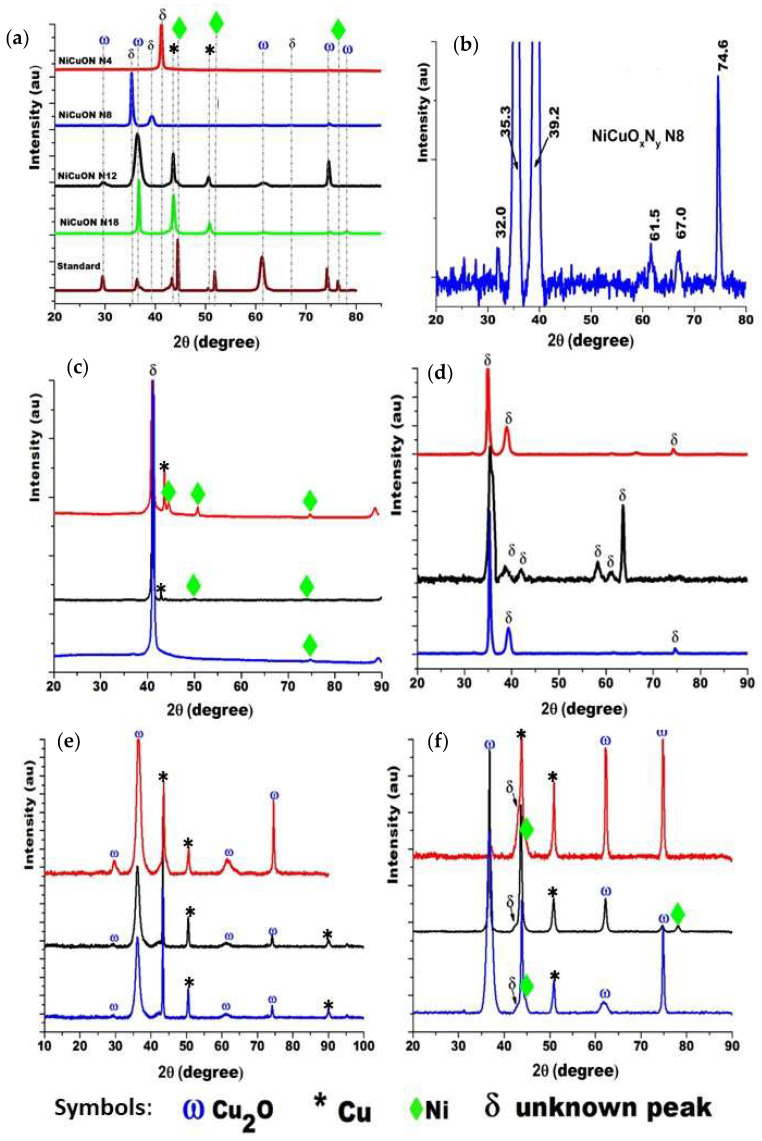
(**a**) XRD patterns of NiCuOxNy deposited under different nitrogen flow conditions. (**b**) Zoom-in for the XRD NiCuOxNy N = 8.00 sccm coating. Triplicate for nitrogen flow: (**c**) 4.00 sccm, (**d**) 8.00 sccm, (**e**) 12.0 sccm, and (**f**) 18.0 sccm.

Figure 5a presents XRD patterns of Cu- and Ni-based coatings grown under varying nitrogen fluxes (with fixed oxygen flux) at a power of 250 W and 433 K. An additional diffractogram of a sample named “Standard” is included as a reference, containing some oxide phases identified in the samples but not oxynitrides, as it was not exposed to nitrogen during preparation. Figure 5b shows an enlargement of the diffractogram obtained for the samples deposited with a nitrogen flow of 8.00 sccm, and Figure 5c–f show the triplicate obtained for each nitrogen flow. For the coating deposited with a 4.00 sccm nitrogen flow, a single peak is observed at 2θ = 41.3°. At 8.00 sccm nitrogen flow, peaks appear at 2θ = 32.0, 35.3, 39.2, 61.9, and 68.4 degrees. Notably, the peaks at 35.3° and 39.2° are 0.1 degrees lower than expected for Cu_2_O, suggesting an increase in interplanar distance. Coatings deposited with nitrogen flows of 4.00 and 8.00 sccm do not correspond to standard copper or nickel oxides or nitrides; hence, these crystallographic phases are denoted as NiCuO_x_N_y_, supported by XPS confirmation of nitrogen presence. For nitrogen flows of 12.0 and 18.0 sccm, the monoclinic copper oxide (Cu_2_O) phase is present, identified with the JCPDS 00–001–1142 pattern card. Additionally, planes (111) at 2θ = 43.5° and (200) at 2θ = 50.6° characteristic of metallic Cu (JCPDS 00–001–1241) are observed. In XRD, planes of nickel oxides are not evident, suggesting an amorphous growth. These results suggest that increased nitrogen flux induces collisions among nitrogen and oxygen molecules, reducing the half-free path and favoring oxygen to reach the substrate, leading to the predominance of copper oxide (Cu_2_O) formation, while some metal remains unreacted.

### 3.3. Morphology

**Figure 6:**
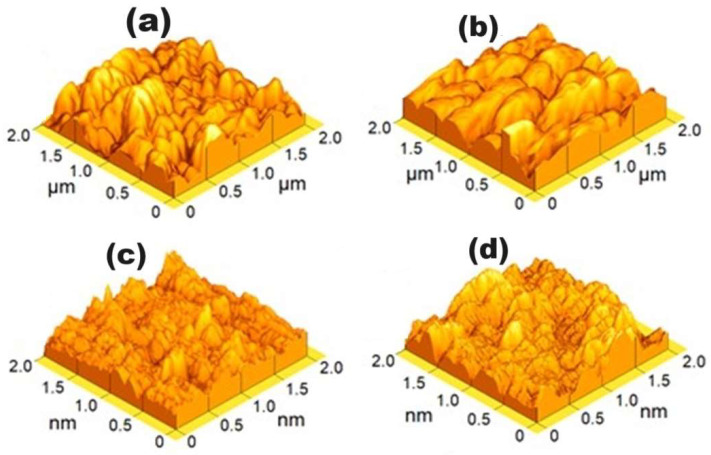
AFM measurements of NiCuO_x_N_y_ coatings. (**a**) ϕN_2_ = 4.00 sccm; (**b**) ϕN_2_ = 8.00 sccm; (**c**) ϕN_2_ = 12.00 sccm; (**d**) ϕN_2_ = 18.00 sccm.

**Figure 7:**
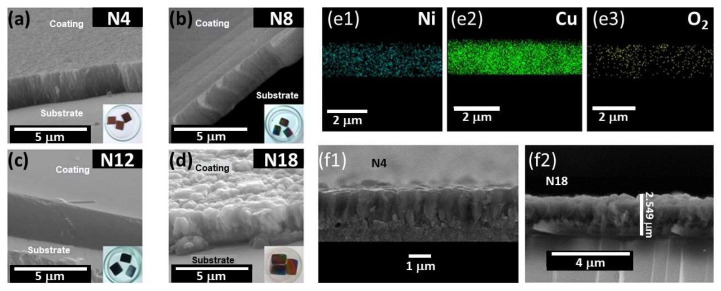
(**a**–**d**) SEM images NiCuO_x_N_y_ N4, N8, N12, and N18 coatings, respectively. (**e1**–**e3**) SEM images in cross-section and EDX mapping for a representative sample: Ni, Cu, and O_2_. Morphology of deposited films showing equiaxial growth with nitrogen flows (**f1**) 4.00 sccm and (**f2**) 18.0 sccm.

AFM and SEM studies support this hypothesis, as depicted in Figure 6 and Figure 7, illustrating the morphology of the coatings under various nitrogen fluxes. Figure 6 reveals that the coating surface appears dense, comprising grains of varied dimensions dependent on the nitrogen flow during growth. Table 3 provides a quantification of grain size and roughness. Results indicate that coatings grown with higher nitrogen flux exhibit smaller grain sizes and lower arithmetic roughness values. Additionally, SEM images (see Figure 7) demonstrate that films deposited at nitrogen flow of 18.0 sccm show equiaxial growth, while those deposited at lower fluxes exhibit columnar growth. These results suggest that the chamber atmosphere’s chemical composition significantly influences film growth mechanisms, thus determining morphology. While a comprehensive investigation into the influence of nitrogen–oxygen mixture and residual gas presence in the chamber exceeds this paper’s scope, the work by Liu et al. (2024) [35] explained the residual oxygen’s effect on metallic film morphology.

The chemical compositions of the coatings in cross-section were determined via SEM/EDX. Figure 7(e1–e3) include representative elemental maps, with results summarized in Table 4. Chemical analysis indicates that nitrogen content falls below the microprobe’s detection limit of EDX, except for coatings deposited at high nitrogen flux. However, XPS results confirm nitrogen presence on the surface of all four coatings. In Figure 7(f1,f2), the morphology shows that the films deposited with nitrogen fluxes of 4.00 sccm and 18.0 sccm present equiaxed growth, while in those deposited with ϕ 8.00 sccm and 12.0 sccm, the growth is columnar.

### 3.4. Optical Properties

Copper- and nickel-based coatings were characterized by having a thickness of 2.43–4.30 μm, with crystallographic phases identified as Cu_2_O and Ni for coatings deposited at higher nitrogen fluxes (18.0 and 12.0 sccm), and an unreported phase, NiCuO_x_N_y_, observed at lower flows (4.00, 8.00, and 18.00 sccm). This investigation evaluated optical properties—refractive index, absorption coefficient, Urbach energy, and optical bandgap energy of NiCuO_x_N_y_ coatings as a function of nitrogen flux and Ni-Cu composition. The results could be used to evaluate the optical functionality, such as the filter of visible wavelengths of the electromagnetic spectrum.

**Figure 8:**
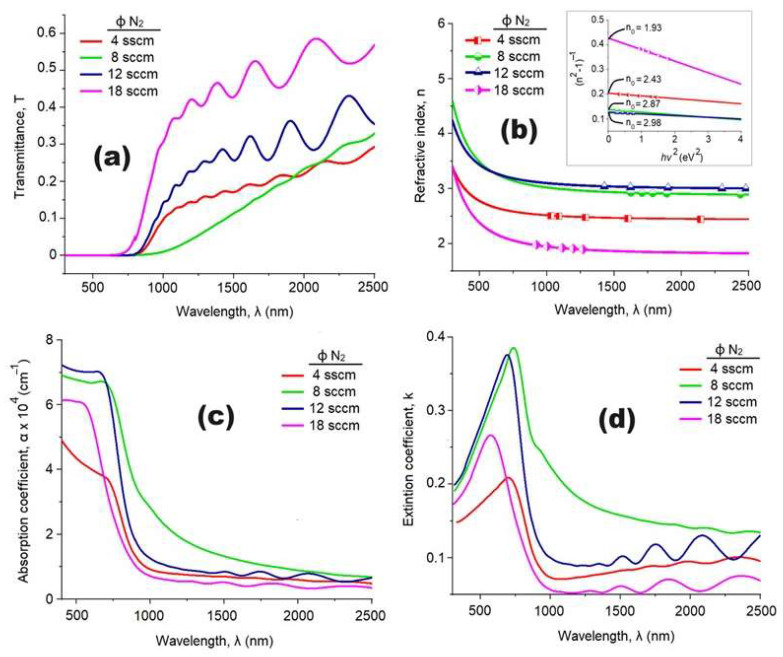
Determination of the NiCuOxNy coating’s optical properties: (**a**) the transmittance spectra obtained for the NiCuOxNy coatings, (**b**) values of refractive index, η, (**c**) absorption coefficient, α, and (**d**) extinction coefficient, k.

Analysis of the transmittance spectra (refer to Figure 8a) confirms that the NiCuO_x_N_y_ coatings effectively block wavelengths below 700 nm. Additionally, coatings produced with a high nitrogen flux exhibit interference patterns indicative of increased thickness (measured in nanometers). Specifically, coatings grown with a nitrogen flux of 18 sccm demonstrate maximum transmittance values ranging between 30% at 700 nm and 60% at 2500 nm. These transmittance values vary with the nitrogen flux; coatings grown at 4 and 8 sccm nitrogen flux exhibit reduced transmittance to 30% at infrared wavelengths. This behavior can be attributed to the surface roughness of the coatings, as films with higher roughness tend to display more pronounced dispersion phenomena at longer wavelengths.

In Figure 8b, starting from approximately 750 nm, the refractive index of all coatings does not exhibit dispersive behavior, as the refractive indices remain constant. These results indicate that the coating with the greatest thickness (N18), characterized by its porous topography and lowest mass density, exhibits the lowest refractive index value, whereas the N8 coating, with lower mass density and low porosity, demonstrates a higher refractive index. This behavior aligns with classical linear optics principles, which predict that the refractive index relies on the porosity and mass density of coatings. Additionally, N12 exhibits a high refractive index, likely due to its dense morphology, motivated by the increased mass density resulting from the presence of unknown crystalline phases within the coating.

On another note, employing well-established expressions A + T + R = 1 and α = (2.303 × A)/t [24,25], where A represents absorbance, T transmittance, R reflectance, α absorption coefficient, and coating thickness, the absorption coefficient was determined as a function of wavelength (refer to Figure 8c). The highest absorption coefficient value was observed for the N12 coating (7.6 × 10^4^ cm^−1^), characterized by a well-defined absorption edge at 980 nm. The remaining coatings exhibited high absorption coefficients ranging from 400 to 1000 nm, after which the absorption coefficient began to decrease. In [48], it was established that absorption coefficient values on the order of 10^4^ cm^−1^ are indicative of increased probabilities of direct transitions between the valence band and the conduction band of semiconductor materials, a prerequisite for considering a material as a viable candidate for photovoltaic applications.

The extinction coefficient (k), calculated as a function of incident radiation wavelength using the expression k = αλ/4π, where λ represents the incident wavelength [24], is shown in Figure 8d. The k value of the N18 coating approaches zero within the wavelength range of 1000–2500 nm, suggesting nearly complete transparency of the coating in this spectral region [40]. Conversely, the N8 coating, possessing the least thickness, exhibits a decrease in its k value from 0.2 at 1000 nm to 0.15 at 1500 nm. The other two coatings demonstrate k-values of approximately 0.1. The transparency of the N18 coating can be attributed to its microstructure, primarily composed of copper oxide, as observed in previous studies [41], while the absorption tails observed in the other films likely result from the presence of crystalline copper nitrides and amorphous oxynitrides of copper.

**Figure 9:**
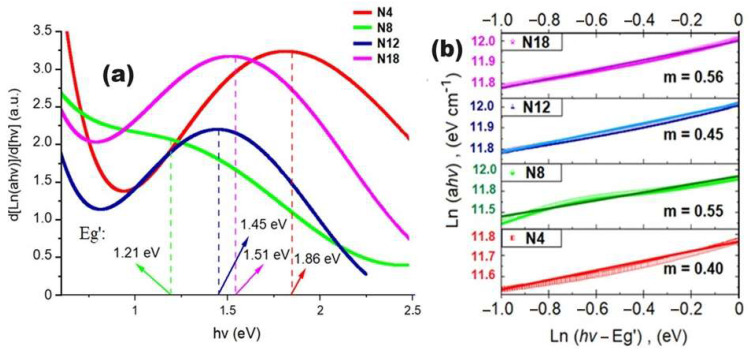
Determination of the transition mode of the obtained NiCuO_x_N_y_ coatings. (**a**) Derivative method of the Tauc model where maximum Eg’ values are determined for the subsequent calculation of the transition mode value (m) from the slope of the plot ln(αhυ) vs. ln(hυ − Eg’) for each NiCuO_x_N_y_ coating (**b**).

On the other hand, for an accurate determination of the transition mode (m) of the NiCuO_x_N_y_ coatings, the derivative method of the Tauc model was employed, i.e., d[ln(αhυ)]/d[hυ] vs. hυ [49,50,51], which exhibits a maximum referred to as Eg’ (Figure 9a). Based on the Eg’ values obtained, the value of m was determined from the slope of the plot ln(αhυ) vs. ln(hυ − Eg’) (Figure 9b). A value of m = 0.5 was found for the NiCuO_x_N_y_ coatings, indicative of an allowed direct transition mode.

**Figure 10:**
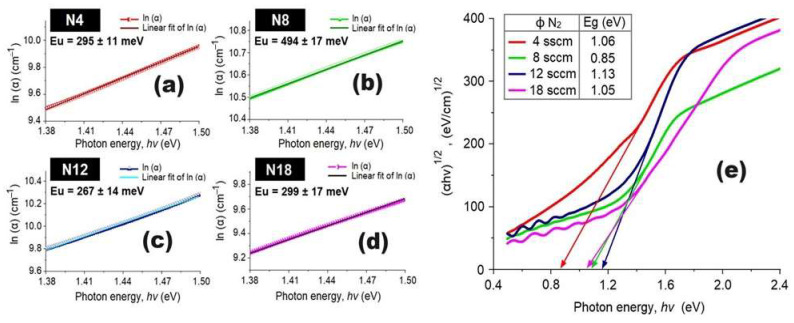
Determination of the Urbach energy (Eu) of the NiCuO_x_N_y_ coatings deposited under different nitrogen flows: N4 (**a**), N8 (**b**), N12 (**c**), and N18 (**d**). Use of the Tauc method for the determination of the optical band gap (Eg) values of the NiCuO_x_N_y_ coatings (**e**).

Considering the optical absorption results, the Urbach energy (E_u_) and optical band gap (E_g_) values were determined. Urbach energy, also known as the Urbach tail, characterizes materials with low crystallinity and amorphous nature, representing the degree of localized states between the valence and conduction bands, causing significant changes in the band gap [52,53]. Using the expression ln(α)= 1/Eu × (hυ) + ln(αo) [51], where α is the absorption coefficient, hυ is the photon energy, and αo is a constant, the value of Eu can be determined by plotting ln(α) vs. hυ. The inverse of the slope corresponds to the value of Eu (Figure 10a–d). Figure 10 shows Urbach energy values at different nitrogen flow rates. A proportional relationship is observed between Eu and the roughness parameters (Ra and RMS), consistent with prior studies [53,54]. For instance, the NiCuO_x_N_y_ N8 coatings exhibit higher Eu values, indicating an increase in disorder and defect states in the film texture, correlating with greater roughness.

**Figure 11:**
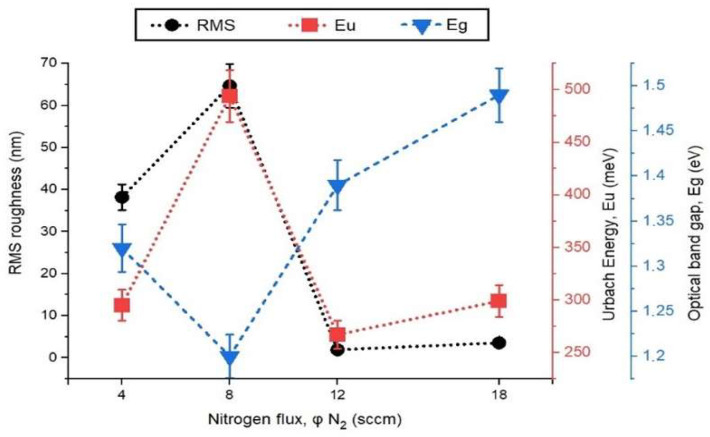
Summarizes the dependence of the RMS roughness parameter and the optical parameters (Urbach energy, Eu, and optical band gap, Eg) on the nitrogen flux during the deposition of NiCuO_x_N_y_ coatings.

Tauc plots were generated to calculate the direct gap, i.e., αhυ vs. β(hυ − E_gap_)^2^, where α is the absorption coefficient, hυ is the incident photon energy, h is Planck’s constant, υ is the frequency of incident light, β is an energy-independent constant, E_gap_ is the optical band gap, and m = 2 represents the previously determined allowed direct transition mode [55]. The results are presented in Figure 10e, where the interception on the energy axis determined that the N18 coating, with the lowest density, has an energy gap of 1.05 eV, while the N4 coating, with the highest density, has an energy gap of 0.87 eV. These results align with classical optics, indicating a decrease in the energy gap with an increase in the mass density of the coatings. Figure 11 summarizes the dependence of the RMS roughness parameter and the optical parameters (Urbach energy, Eu, and optical band gap, Eg) on the nitrogen flux during the deposition of NiCuO_x_N_y_ coatings. In Figure 11, the relationship among RMS roughness, Urbach energy (Eu), and the energy gap of NiCuO_x_N_y_ coatings deposited at different nitrogen fluxes (4, 8, 12, and 18 sccm) is shown. The results indicate that the roughness decreases from 67.4 nm (8 sccm) to 3.55 nm (18 sccm). These results could be explained by considering a better incorporation of nitrogen into the crystallographic structure of the coatings. In general, the Urbach energy values indicate that the coatings do not have impurities in their electronic structure. Finally, the energy gap of the coatings indicates that the nitrogen flux does not strongly affect this gap.

## 4. Conclusions

In this comprehensive investigation, we explored the deposition and characterization of NiCuO_x_N_y_ coatings prepared via RF-magnetron sputtering on common glass substrates. Through XPS analysis, we examined the surface chemistry of the coatings, revealing the presence of copper oxide, copper nitride, and copper oxynitride species. The XRD analysis further unveiled crystalline phases not previously documented in existing databases or the scientific literature, underscoring the novelty of the synthesized materials.

Our optical studies provided valuable insights into the coatings’ behavior across the UV-VIS electromagnetic spectrum. By systematically varying the nitrogen flux during deposition, we observed notable changes in the density of states between the valence and conduction bands, elucidating the observed increase in absorption coefficient with higher nitrogen flux. This phenomenon highlights the tunability of the coatings’ optical properties, offering potential applications in optical filtering and light absorption technologies.

Furthermore, our investigation into the energy band gap of the coatings revealed semiconductor-like behavior, with energy gaps ranging from 1.21 to 1.86 eV. This finding underscores the potential of NiCuO_x_N_y_ coatings in photovoltaic applications, where semiconductor materials with suitable band gaps are crucial for efficient solar energy conversion and optical filters. Future research efforts could delve deeper into optimizing deposition parameters to tailor the properties of these coatings for specific application requirements.

## Figures and Tables

**Table 1:** Deposition conditions of NiCuO_x_N_y_ coatings.

Sample	Sputtering Deposition Parameters			
	ϕN_2_ (sccm)	Power (W)	T (K)	Cu/Ni Ratio in Target
NiCuO_x_N_y_ N4	4.00	250	433	6.14
NiCuO_x_N_y_ N8	8.00			
NiCuO_x_N_y_ N12	12.00			
NiCuO_x_N_y_ N18	18.00			

**Table 2:** Composition of NiCuO_x_N_y_ coatings.

Coating Sample	ϕN_2_ (sccm)	BE N1s (eV)	Chemical Formula
NiCuO_x_N_y_ N4	4.00	400	CuNi_0.2_O_0.93_N_0.09_
		397	CuNi_0.2_O_0.93_N_0.12_
NiCuO_x_N_y_ N8	8.00	400	CuNi_0.4_O_0.8_N_0.8_
		397	CuNi_0.4_O_0.9_N_0.7_
NiCuO_x_N_y_ N12	12.0	400	CuNi_0.2_O_0.5_N_0.03_
		397	CuNi_0.2_O_0.4_N_0.03_
NiCuO_x_N_y_ N18	18.0	400	CuNi_0.2_O_0.7_N_0.04_
		397	CuNi_0.2_O_0.7_N_0.04_

**Table 3:** Roughness and thickness of NiCuO_x_N_y_ coatings.

Coating Sample	Average Roughness Parameters (Mean ± SD)		Thickness
	RMS (nm)	Gran Size (nm)	μm
NiCuO_x_N_y_ N4	38.20 ± 0.86	131.00 ± 9	2.43
NiCuO_x_N_y_ N8	64.70 ± 0.95	252.00 ± 14	2.50
NiCuO_x_N_y_ N12	1.93 ± 0.07	9.00 ± 1	4.30
NiCuO_x_N_y_ N18	3.55 ± 0.03	14.00 ± 1	2.86

**Table 4:** Composition of NiCuO_x_N_y_ coatings in cross-section via EDX.

Coating Sample	Element wt (%)			
	Ni	Cu	O_2_	N_2_
NiCuO_x_N_y_ N4	6.27	90.3	3.45	ND
NiCuO_x_N_y_ N8	5.42	92.3	2.26	ND
NiCuO_x_N_y_ N12	5.35	85.3	4.13	5.18
NiCuO_x_N_y_ N18	6.51	81.0	3.93	8.53

## Data Availability

The original contributions presented in the study are included in the article, further inquiries can be directed to the corresponding author.
